# Whether the digital divide widens the income gap between China’s regions?

**DOI:** 10.1371/journal.pone.0273334

**Published:** 2023-02-13

**Authors:** Ying Qiu, Nianci He, Chenjing Yan, Qiao Rao

**Affiliations:** 1 School of Economics, Jiangxi University of Finance and Economics, Nanchang, Jiangxi Province, China; 2 School of Economics and Management, China University of Geosciences (Wuhan), Wuhan, Hubei Province, China; Universiti Malaysia Sabah, MALAYSIA

## Abstract

Based on the panel data of 280 prefecture-level cities in China from 2014 to 2018, we construct the digital divide index from three aspects including "access divide", "use divide" and "efficiency divide", using Thiel index to measure income inequality and study the impact of the digital divide on income inequality between different regions. We apply the two-stage spatial DID combined IV model. In the first stage, we introduce the "Internet + government affairs" policy to construct the spatial DID model, so as to obtain the estimated value of the digital divide index. When the estimated value is substituted into the two-stage regression as an instrumental variable, it is found that every 1unit increase in the digital divide will widen the income divide by 0.134 units. This conclusion is still robust under the replacement of Theil index and change of weights. The results of heterogeneity analysis show that digital divide has a more significant impact on the income divide in eastern China in terms of regions. It is found after distinguishing different types of digital divide that the other two types of digital divides have the most significant impact than income divide, 0.034 units higher than that of the traditional. Finally, we introduce the mechanism variables from three levels of "access divide", "use divide" and "efficiency divide", thus verifying the effect of information asymmetry, effect of human capital differentiation and effect of delaying industrial upgrade. Then we put forward the following policy recommendations: in order to reduce the deterioration effect of digital divide on income distribution, it is necessary to improve infrastructure, enhance the degree of digitalization of human capital and optimize the industrial structure.

## I. Introduction

Recent years have witnessed the rapid development of digital economy, which is new impetus to the economic transformation and upgrade. However, due to the differences between resource endowment and use efficiency, digital transformation objectively widens the digital divide. The digital divide is manifested in the disparities in Internet infrastructure access between different regions, the differences between the ability and frequency of individuals to use the Internet, and the differences in the returns brought about by digital technologies in different regions. Its existence further enlarges the endowment of information resources in different regions. As a factor of production, information continues to increase its participation in the economy, which makes the influence of digital divide on the income divide between regions keep growing. Recent literature rarely quantifies the digital divide and mostly analyzes the negative impact of the digital divide on society from a qualitative perspective.

We discuss the influence and mechanism of digital divide on income divide in a theoretical and empirical way. Firstly, based on the practice of Xu Xiang et al. [1], we introduce the production function including data capital, and compare the difference of growth rates between different types of capital under steady-state conditions, finding that data capital grows the fastest. The existence of digital divide makes the shares of data capital used by different regions vary greatly, which will eventually widen the income divide between regions by affecting the level of regional output. Secondly, relied on the panel data of prefecture-level cities from 2014 to 2018, we construct the digital divide index from three levels of "access divide", "use divide" and "efficiency divide", and utilize the two-stage spatial DID+IV model, so as to empirically test the impact of digital divide on income divide. The "Internet + government affairs" policy is introduced into the first-stage regression to construct the spatial DID model, and the exogenous digital divide index estimate is obtained, which is used as tool variable to deal with the endogenous problems. By introducing different mechanism variables from the three dimensions of "access divide", "use divide" and "efficiency divide" respectively, we verify the three ways that digital divide affects income distribution. The results of this paper remain robust under the regression of benchmark model, heterogeneity analysis of different regions, types of digital divide and robustness test. Firstly, the existence and expansion of digital divide will further widen the income divide between regions. Secondly, the digital divide in the eastern and coastal areas has more significant influence on income divide, and the new digital divide has more significant influence than the traditional one. Thirdly, the digital divide can affect income distribution through access divide, use divide and efficiency divide, resulting in asymmetric information, differentiation of human capital and sluggish upgrading of industrial structure.

This paper is related to the following three literatures. The first is the study on digital divide, covering definition, measuring method and influence. The concept of digital divide was first proposed by Toffler [2] and further enriched by Norris [3], Mossberger et al. [4] and Van Deursen et al. [5]. Digital divide has gradually expanded from simply divide in Internet infrastructure construction between regions to the income inequality brought by the Internet. Currently, the measurement methods of digital divide mainly include single-index measurement and multi-index measurement, the former one refers to the margin ratio method. The key of multi-index measurement lies in determining the weight to get the relevant indexes (Xiao and Wu, [6]). Scholars have deeply analyzed the digital divide from the perspective of delaying the upgrading of industrial structure, influencing the relative sense of separation of micro-individuals and widening the income divide between urban and rural areas (Chen et al., [7]; Luo and Cha, [8]; Peng et al., [9]; Zhang, [10]; Kong et al., [11]).

The second part is the study on income divide, containing measurement methods, development trends and influencing factors. The research on the measurement of income disparity began earlier. Sen et al. [12] systematically expounded the conceptual framework and the practical problems of inequality measurement, and proposed an improved method of Gini coefficient to measure inequality. Shorrocks [13] proposed generalized entropy index based on Thiel index. Scholars often used urban-rural income ratio, Gini coefficient and Thiel index to measure inter-regional income inequality (Zhao, [14]; Chen and Li, [15]). In addition, it is found that the income divide in China shows that: 1. In terms of distribution pattern, it shows typical spatial heterogeneity (Tian, [16]; Li and Wang, [17]). In terms of the development trend, the income divide continues to rise, but there is a convergence trend (Knight et al., [18]; Li and Zhu, [19]). The existing researches mainly analyze the influencing factors of income divide at the macro level, such as democracy, financial development and globalization (Acemoglu et al., [20]; Jauch and Sebastian, [21]; Dorn et al., [22]).

The last part is the study on the influence of digital technology on income distribution. First of all, digital technology can improve the information asymmetry and reduce the income divide between regions by popularizing the Internet (He and Xu, [23]). Secondly, digital technology can change the structure of factor allocation, Benzell et al. [24] think that the digitization trend can reduce the marginal cost of labor and capital, but result in highly unequal rewards for talents lacking supply elasticity. In addition, Korinek et al. [25] think that the use of artificial intelligence technology will lead to a further widening of the skill divide among workers, which further enlarges the inequality in income distribution.

The possible marginal contribution in this paper lies in: First, we make exponential innovation to quantify the digital divide. We construct the digital divide index from three levels: "access divide", "use divide" and "efficiency divide", expanding the connotation of the existing indexes. While previous construction of indexes mostly focused on the differences in digital infrastructure, we focus on the "efficiency divide", or the difference in returns brought by digital technology. Secondly, we make method innovation to apply spatial DID + IV model. Based on the counter-fact frame, the exogenous digital divide index is obtained, which reduces the error of parameter estimation caused by endogeneity in the model. Thirdly, we make theoretical model innovation to introduce data capital-contained production function, and use the growth rate difference of different capital to reflect the widening effect of digital divide on income divide.

The structure of this paper is as follows: The first part is the introduction; the second part is the theoretical modeling and mechanism analysis; the third part is the econometric model and variable explanation; the fourth part is the regression result and analysis; the fifth part is the conclusion and policy suggestion.

## II. Theoretical mechanisms

### (I) Information asymmetry caused by access divides

The access divide mainly refers to the difference in information infrastructure construction, which will hinder the normal information exchange between regions. Firstly, the access divide brings information cost effects. The region with complete information infrastructure construction has strong network effect, and the searching cost of digital commodity information is close to zero. However, the regions with low construction level are faced with information barriers, which makes it difficult to obtain effective information resources at low cost. The difference between information search and access costs will further widen the income divide between regions.

Secondly, the access divide makes the supply and demand of information mismatched. The economic development of backward regions is slow and advanced knowledge and technology is required to be introduced to adjust the solidified industrial structure. However, the construction level of information infrastructure reduces the accessibility of information, and it is not easy for the backward regions to obtain accurate information suitable for local development level, which leads to the stagnation of their development level. While developed regions can receive abundant information resources from both domestic and foreign countries, resulting in information asymmetry among regions, which will widen the income divide between regions.

Thirdly, the policy of promoting "new infrastructure" is mostly piloted in regions with relatively complete information infrastructure, while the policy, as a kind of institutional supply, will enlarge the existing infrastructure advantages of developed regions. Compared with traditional infrastructure, the core digital technology of "new infrastructure" is more extensive, innovative and obvious spillover effect, and the digital products formed by it have the characteristics of zero marginal cost or close to zero in production. The advanced regions will carry out digital reconstruction for the traditional infrastructure through "new infrastructure" to improve the multiplier effect of traditional infrastructure on economy, while the backward regions can’t enjoy the policy dividend, and it is quite hard to build complete information infrastructure, causing that construction divide of information infrastructure will eventually widen the income divide between regions. Additionally, featured by spatial spillover effect of new infrastructure policy, the advanced regions take the lead in building the new infrastructure construction, driving the development of the surrounding regions. However, due to the access divide, it is difficult for the surrounding regions to receive relevant knowledge and technology, which weakens the spillover effect of policies, and finally leads to the widening of the income divide between regions.

### (II) Effect of human capital differentiation caused by use divide

The use divide mainly refers to the difference between people’s ability and frequency of using Internet, which will lead to obvious differentiation of human capital, one is digital human capital, and the other is traditional human capital. According to the new human capital theory, the ability factors (including cognitive and non-cognitive abilities) that are not easily replaced by technological factors are the key factors influencing income inequality. Firstly, compared with traditional human capital, digital human capital can promote laborers’ income through information channel effect, financing effect and social interaction effect, thus widening the income divide between the two kinds of human capital.

Secondly, as for the inside of digital human capital, informatization and digitalization will produce technology-biased progress, bring polarization effect to labor market, increase relative demand for high-skilled labor force, and induce skill premium phenomenon. And the huge benefits brought by digital non-competition are highly concentrated in the hands of the executives and shareholders of the large digital enterprises, which belong to the high-skilled management level within the digital human capital. However, the workers of other executive levels can only share the surplus income together, thus divide is applied to generate a larger income divide within the digital human capital.

Thirdly, Internet policy can indirectly influence regional innovation ability by promoting the inter-regional flow of innovation capital and innovators. Moreover, the developed regions can fully combine local advantages and policy dividends, which can produce huge siphon effect, further drain the innovative human capital in backward regions, and further widen the divide of innovation capacity between different regions. What’s more, innovation can widen the income divide through "productivity effect" mainly caused by that the developed regions can take advantage of innovation more effectively to improve local productivity, that is, the promotion on productivity of developed regions by innovation is greater. Besides, the implementation of Internet policy has spillover effect on the improvement of innovation level in surrounding regions. However, due to the existence of use divide, the surrounding regions may not possess the literacy and ability to accept relevant innovation knowledge, resulting in differentiation of innovation level among regions, and finally widening the income divide between regions.

### (III) Delayed effect of industrial upgrading brought by benefit divide

The benefit divide mainly refers to the difference in returns brought by digital technology, which will slow down the industrial structure in backward regions and upgrade income divide between regions. Firstly, reduce industrial efficiency and delay the integration of informatization and industrialization. Hindering the integration of informatization and industrialization will not be conducive to the emergence of efficient technological means, mode of production and technological form, thereby reducing industrial efficiency. While the improvement of industrial efficiency is the main way to promote the upgrading of industrial structure, and the existence of benefit divide will delay the optimization and upgrading of industrial structure in backward regions, that not only enlarges the existing endowment difference, while boosting the probability of element mismatch, which will lead to the widening of income divide between regions.

Secondly, the benefit divide is not conducive to the combination of industry and finance, and reduces the efficiency of capital allocation. The level of financial digitalization varies greatly in different regions. The traditional financial sector faces the dilemma such as "domain mismatch" and "scale mismatch". Local enterprises are still constrained on financing issues, which is not favorable to realizing the combination of industry and finance, debasing the speed of digital transformation and blocking the upgrading of industrial structure. Enterprises in developed regions can timely obtain sufficient funds for innovation activities such as R & D, so as to promote the upgrading of the overall industry, and thus obtaining higher profits. The ultimate benefit divide will widen the income divide between regions and enterprises by affecting the speed of financing.

**Fig 1:**
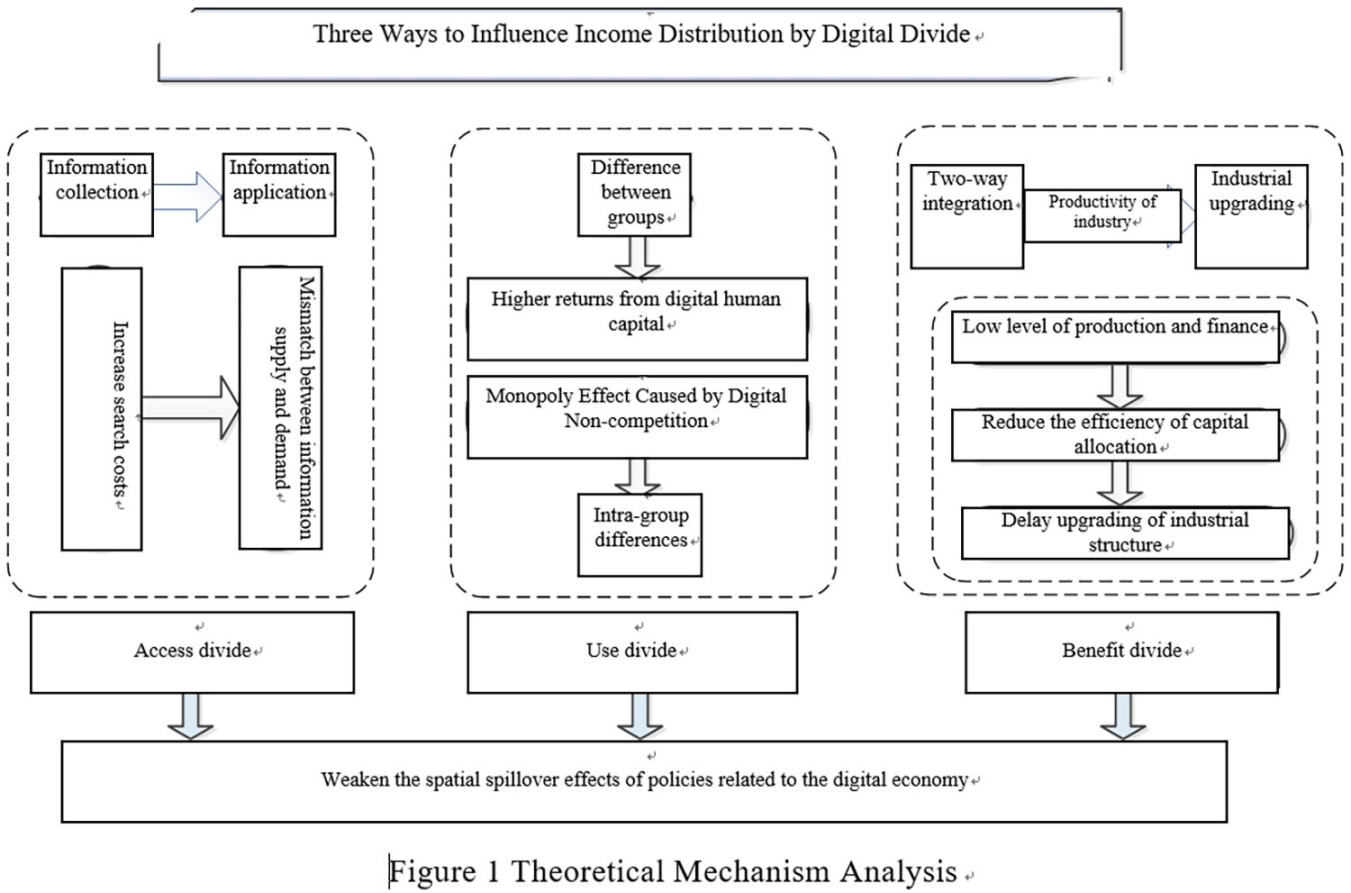
Flowchart on theoretical mechanisms. Source: Drawn by authors.

Thirdly, the policy for promoting the industrial digitalization transformation can advance the cross-border integration of industry, reconstruct the competition mode of industrial organization, and upgrade the industry power. Due to the fact that the industrial base in backward region is weak, so it is difficult to realize digital transformation in a short time by the assistance of policies, on the contrary, advanced regions can fully combine technology and policy dividend to steadily promote industry digitization which can bring huge multiplier benefit to economy, and become the main engine of driving economic development day by day. Differences in industry digitization levels between different regions will further widen the income divide. As an important part of digital economy, industrial digitalization has significant spatial spillover effect, which offers support in improving the total factor productivity of neighboring regions. As a result of the existence of benefit divide, it is difficult for neighboring regions to undertake digital industries in advanced regions, and the promotion of total factor productivity is limited, bringing about productivity differences among regions, and eventually inducing income inequality (Fig 1).

## III. Empirical methodology

### (I) Model construction and data description

#### 1. Model construction

In order to verify the impact of the digital divide on income distribution, the following model is developed with the use of panel data from prefecture-level cities from 2014 to 2018:

$${\mathrm{theil}}_{it}=\alpha_{0}+\beta_{1}gap_{it}+\beta_{2}\sum X_{it}+\eta_{i}+\xi_{it}$$ (1)

Where i represents region and t represents year; and *theil*_*it*_ represents the Thiel index for measuring income disparities between different regions. *gap*_*it*_ represents the digital divide index, *X*_*it*_ represents the control variables in the model, including the regional control variables: economic openness (*open*_*it*_), urbanization level (*urbanize*_*it*_), industrial level control variables: industrial structure deviation (*stru*_*dev*_*it*_), and individual level control variables: logarithm of per capita real GDP (ln*gdppc*_*it*_), average years of education (*edu*_*it*_), and dependency ratio with the elderly (*dependency*_*it*_).

Considering that the digital divide index is not an exogenous variable, and there is has a causal relationship between the digital divide index and income divide, we further establish a spatial DID+IV model to deal with the endogenous problem. Since the spillover effect between the control groups is not obvious, the spillover effect of the experimental group to the experimental group and the experimental group to the control group is considered only. In which *W*_*T*,*T*_ represents the indirect effect of the experimental group to the experimental group, and *W*_*NT*,*T*_ represents the indirect effect of the experimental group to the control group. Instrumental variables have both relevance and exogenous characteristics. On the aspect of relevance, the exogenous policy of "Internet + government affairs" which is closely related to the digital divide has been selected. By using the spatial DID model to carry on the regression and based on the counter-fact frame; it can be known the causal effect of exogenous policy on the digital divide, so the estimated digital divide index also has the exogenous characteristic, which can’t directly affect the income divide. The regression between the estimated value of digital divide index and income divide will result in a robust result, which reduces the deviations of model parameter estimation.


$$gap_{it}=\alpha_{0}+\beta_{1}DID_{it}+\beta_{2}W_{TT}DID_{it}+\beta_{3}W_{NTT}DID_{it}+\beta_{4}\sum X_{it}+\eta_{i}+\xi_{it}$$ (2)

$$theil_{it}=\alpha_{0}+\beta_{1}g\hat{a}p_{it}+\beta_{2}\sum X_{it}+\eta_{i}+\xi_{it}$$ (3)

#### 2. Data description

We select the data of 280 prefecture-level cities in China from 2014 to 2018 as the research samples, and the data in the empirical study mainly come from *“China City Statistical Yearbook”*, *“Statistical Bulletin of National Economic Development”*, *“China Broadband Rate Download Report”*, RESSET Financial Research Database and EPS Database, etc.

### (II) Variable selection and index calculation

#### 1. Exponent measure of digital divide

This article chooses "access divide", "use divide" and "benefit divide" as the first-level indicators to construct digital divide index. The "access divide" refers to the inequality of Internet access, referring to *“China Digital Economic Development Index Report 2017”*, while Internet broadband user access rate and mobile phone penetration rate are selected to reflect the inequality of Internet infrastructure construction. The "use divide" refers to the inequality of Internet use capacity and frequency, referring to the construction of the measurement index systems of *“Digital Divide and Anti-Poverty Research”*, and the Internet penetration rate and broadband download rate are selected to reflect the divide in Internet use process.

"Benefit divide" brings digital industrialization, industry digitization and digital government governance into the index system, reflecting the difference of returns brought by digital factors. Among them, digital industrialization includes the development level of information communication industry such as software and information technology service industry, the income of software and technical service, the income of software product, the number of information transmission and computer service employees, and the number of Taobao Village. The construction of digital government, an important part of digital governance, adopts the influence index measured by *"Influence of Government Affairs Weibo" and "Influence of Government Affairs WeChat"* published by People’s Daily to reflect the level of digital government governance.

In the process of calculating industry digitization, refer to the measurement framework of China Digital Economic Development White Paper [26]. Currently, the basic measurement method of industry digitalization widely used in academic circles is the perpetual inventory method (PIM), which uses the data of fixed assets investment to obtain the capital stock in different years, and then measures the industry digitalization level in different regions (Table 1).

**Table 1:** Digital divide indicator system.

Level I	Level II	
Access divide	Internet broadband access rate	
	Mobile phone penetration rate	
Use divide	Internet penetration rate	
	Wideband download rate	
Benefit divide	Digital industrialization level	Income from software and technical services
		Software Product Revenue
		Information Transmission, Computer
		Number of Service Practitioners
		Number of Taobao Village
	Digital government development level	Government Weibo and WeChat Influence Index
	Digital level of industry	Capital stock measured by perpetual deposit method (refer to CAICT)

Source: Collated by the author.

#### 2. Income divides measurement

The existing methods of measuring income divide mainly include Gini coefficient, Thiel index and urban-rural income ratio. The Gini coefficient is based on assumptions of different population distributions, and required to be derived using micro-individual income data. It is difficult to make panel data because the existing micro-database is investigated at intervals of several years. Therefore, we adopt the Thiel index and the urban-rural income ratio in the process of measuring the income divide. Thiel index applies the concept of entropy to measure income inequality, fully considers the population structure and income change, and complies with the current urban-rural dualistic structure, showing strong practical significance. The calculation method is as follows:

$$theil_{it}=\sum_{j=1}^{2}\left(\frac{Y_{ij,t}}{Y_{i,t}}\right)\ln(\frac{Y_{ij,t}}{Y_{i,t}}/\frac{N_{ij,t}}{N_{i,t}})$$ (4)

Where, *theil*_*it*_ refers to the Thiel index of region i and period t, reflecting the income divide between urban and rural regions. *Y*_*i*,*t*_ represents the total income of the cities and towns (villages) of Region i, *Y*_*i*_ represents the total income of the urban and rural regions of Region i. *N*_*ij*_ represents the total population of cities and towns (villages) of Region i, and *N*_*i*_ represents the total urban and rural population of Region i.

#### 3. Intermediary variable

Post and telecommunication service income (*post*): proxy variable as information asymmetry (Li, [27]); In remote and poor regions, the transportation convenience is insufficient and the communication infrastructure is scarce, so the residents have insufficient ability to obtain information, receive education and medical care, and information development is weak. The foundation of the Internet lies in post and communication. It is necessary to improve the availability of post and telecommunications, so that more people may enjoy related services. Specific measures include increasing the distribution density of postal network points and improving the efficiency of the postal and telecommunications industry. The physical distance between increasing branches of post and telecommunications institutions and customers will be shortened, and the degree of information asymmetry will be alleviated. polarization effect of labor market: namely, the sum of proportion of high-skilled and low-skilled labor divided by the proportion of medium-skilled labor (*laborpolar*) as proxy variable of human capital structure; proportion of employed population of tertiary industry (*tratio*): proportion of employed population of tertiary industry in total labor force, as agent variable of industrial structure.

#### 4. Control variables

The definition and measurement of concerned control variables are as shown in Table 2.

**Table 2:** Control variables and their explanations.

Variable	Variable definition and calculation method
Logarithm of per capita real GDP of the region (*gdppc*)	Based on the calculation of per capita regional GDP index (last year = 100), the fixed base index with 2014 as the base year is obtained, and the per capita GDP of the current year is reduced to obtain the actual GDP per capita of each prefecture-level city. Finally, the logarithmic processing is carried out.
Economic openness (*open*)	By utilizing the ratio of total import and export volume of each prefecture-level city to local GDP, measure the degree of trade dependence is measured
Average number of years of education (*edu*)	$\sum \sum h_{ij}*N_{ij}/N_{j}$, where *h*_*j*_ indicates the number of years of education at different levels in Region i, with 16 years for university education (*h*_1_), 12 years for senior secondary education (*h*_1_), 9 years for junior high school education (*h*_3_), 6 years for primary education (*h*_4_) and 1 year for illiterate population (*h*_5_). *N*_*ij*_ represent the number of people corresponding to the years of education at different levels in Region j, and *N*_*j*_ represents the total population of Region j.
Urbanization level (*urbanize*)	It is measured by the ratio of resident population to resident population in local cities and towns
Deviation degree of industrial structure (*stru*_*dev*)	It is mainly used to calculate the deviation degree between industrial added value and labor employment, which is a negative indicator, namely the higher the industry deviation degree, the more unreasonable the industrial structure. It is calculated as follows: $stru\_dev=\sum_{i=1}^{n}\left|Y_{i}L/YL_{i}\right|$, where *Y*_*i*_ represents the added value of Industry i, of which *L*_*i*_ represents the number of labor force in Industry i. Industrial Deviation Degree is the Control Variable at Industry Level
Elderly dependency ratio (*dependency*)	It eflects the age structure of different regions, and use this indicator to control factors at the age level

Source: Collated by the author.

#### 5. Instrument variables

It is generally believed that there is an endogenous problem between the digital divide and income distribution, and the digital divide will affect the income distribution. Additionally, the digital level of the regions with higher income level is high, while the digital development level of the regions with lower income level is slow, that is to say, there may be a causal relationship between the digital divide and income distribution. We will adopt the instrument variable method to solve the endogenous problem. Instrument variables must have the following two characteristics: relevance and externality. On the one hand, the instrument variable selected is closely related to the digital divide, on the other hand, it is irrelevant to the error term, that is, the instrument variable cannot directly affect income distribution.

We selects the estimation term of X as instrument variable to be endogenously processed, therefore, we use the national policy "*Promoting the ‘Internet plus Government Services’ to carry out the pilot implementation plan of information benefiting the peopl*" issued by the National Development and Reform Commission of China in 2016 as an exogenous variable. The specific connotation of this policy is to select 80 pilot cities with both their urban and rural areas in China, building up the integrated online government service platform, realizing the standardization of government services by disclosing government service matters to all urban and rural residents within the jurisdiction.

The policy is implemented at the prefecture-level city level, covering all urban and rural areas in 80 pilot cities, namely Shenzhen, Jinan, Yinchuan, Foshan, Weifang, Jiaxing, Suzhou, Yingtan, Benxi, Wuhu, Changchun, Urumqi, Guangzhou, Siping, Chengdu, Wenzhou, Weihai, Linfen, Xiamen, Fuyang, Guiyang, Dongguan, Fuzhou, Quanzhou, Chongqing, Dalian, Golmud, Xianyang, Luoyang, Daqing, Hangzhou, Xiaogan, Yiyang, Xi’an, Xinyu, Shenyang, Liaoyuan, Huaian, Qinhuangdao, Hohhot, Qingdao, Yuxi, Wuxi, Guilin, Shizuishan, Ningbo, Zhengzhou, Yuncheng, Yichang, Baiyin, Beijing, Wuzhou, Nanning, Shihezi, Shanghai, Jiyuan, Xiangtan, Shangrao, Neijiang, Chengde, Wuhai, Qitaihe, Lhasa, Tianjin, Lanzhou, Baoshan, Hefei, Shijiazhuang, Alar, Putian, Wuzhong, Karamay, Mianyang, Xiangyang, Wenshan, Changsha, Liaoyang, Yining, Harbin, Dunhuang.

First, to bridge the access divide by building an Internet government affairs platform and alleviating the information asymmetry level of all urban and rural residents within the areas; Second, to reduce the use divide by increasing the inclusiveness of government services to reduce the degree of human capital differentiation; Third, to bridge the benefit divide by improving the intelligence of government services horizontally reduce the transaction costs of enterprises as market entities. Therefore, we selected 2016 as the event shock year, the urban and rural areas of pilot cities as the experimental group, and the urban and rural areas of non-pilot cities as the control group to establish a spatial DID model with two-stage regression.


$$gap_{it}=\alpha +\beta_{1}DID_{it}+\beta_{2}W_{TT}D_{it}+\beta_{3}W_{NTT}D_{it}+\beta_{4}edu_{it}+\beta_{5}stru\_dev_{it}+\eta_{i}+\xi_{it}$$ (5)

On the basis of policy externality, the spatial DID model constructs a counter-fact frame and the regression coefficient can reflect the causal effects of the policy of “"Internet + government affair”" on the digital divide. Therefore, the estimated value of digital divide index obtained is exogenous, and it can’t directly affect income divide.

## IV. Benchmark regression results and analysis

### (I) Descriptive statistics

The descriptive statistics has been shown in Table 3 as follows:

**Table 3:** Descriptive statistics results.

Variable	N	mean	sd	min	max
theil	1400	0.1126	0.1452	1.74e-07	1.0208
gap	1400	10.367	13.3553	1.1396	174.144
open	1400	0.0179	0.0728	0	1.3316
edu	1400	9.0179	0.5261	7.5138	12.675
alntfp	1400	8.0464	0.8821	4.8929	11.7144
tratio	1400	53.8329	13.565	23.6582	90.1
lnpost	1400	10.7945	1.2356	7.2079	15.8418
urbanize	1400	0.5458	0.1519	0.1397	1.0387
stru_dev	1400	49.021	106.29	0.0506	1648.77
lngdppc	1400	10.755	0.5268	9.2273	12.3201
dependency	1400	36.168	5.9682	23.02	50.56

Source: Calculated by the author.

### (II) Spatial DID model

We use the policy of “*Internet plus Government Services*” which was proposed in 2016, so year 2016 was selected as the event impact year. Choosing pilot cities as experimental group and non-pilot cities as control group to establish the spatial DID model under bi-directional fixed effect, and this model can get the net effect of policy through the difference of two times, which can truly reflect the influence of policy.

Traditional DID models assume that the potential outcome of any individual will not be affected by other individuals, which is called the Stable Unit Processing Value Hypothesis (STUTVA). However, in practice, the neighbor regions will also influence the local region, so using the traditional DID model may lead to error in parameter estimation. SDID will be more effective in estimating policy effects, so the model is further expanded and the specific expression of the model is as follows:

$$gap_{it}=\phi_{i}+\theta_{t}+\mu (X_{it})+\alpha DID_{it}+\beta_{1}W_{TT}DID_{it}+\beta_{2}W_{NTT}DID_{it}+\xi_{it}$$ (6)

In which, *W*_*T*,*T*_ represents the indirect effect of the experimental group to the experimental group, *W*_*NT*,*T*_ represents the indirect effect of the experimental group to the control group. *α* refers to the direct impact of Internet + government policy on the digital divide in this region, *β*_1_ represents the spillover effect of experimental group to neighboring experimental groups, and *β*_2_ represents the spillover effect of experimental group to neighboring control groups.

#### 1. Parallel trend test

**Fig 2:**
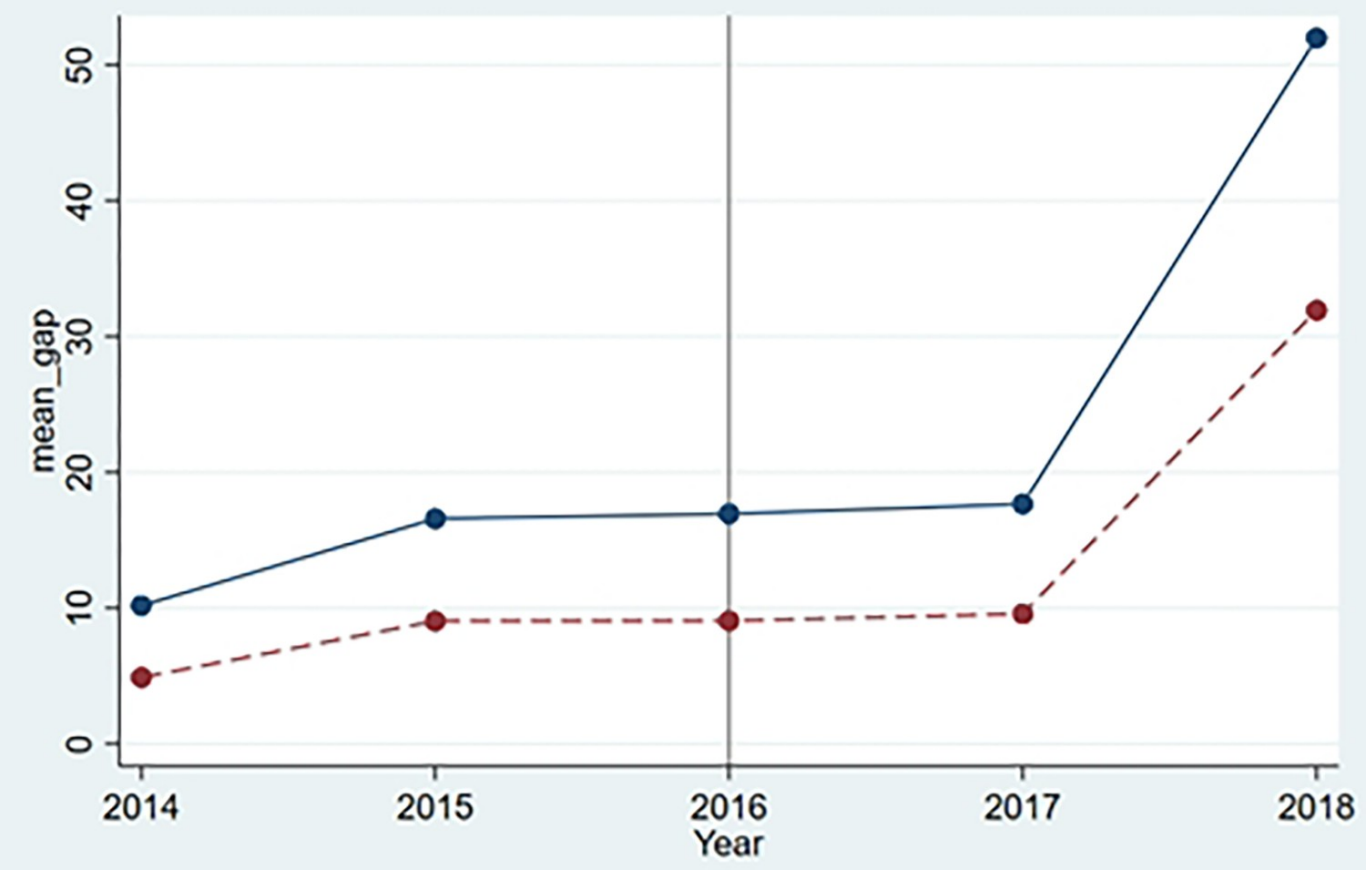
Parallel trend test. Source: Drawn from stata 16.

Before the double difference model can be tested empirically, a parallel trend test must be carried out. The implication of this test is that the experimental group and the control group must have the same trend before and after the implementation of the policy. DID model allows for some differences between the control group and the processing group, provided that the difference does not change over time. The common trend test can be done either graphically or empirically. Considering the non-experimental period before 2016, the digital divide index is regressed to exogenous variable X, time trend variable, interaction term between time trend variable and group virtual variable as well as group virtual variable. The final result showed that the coefficient of interaction term was not significant, P value was 0.874, and the parallel trend test could be judged as qualified by combining graph and regression results. The parallel trend test shows that the policy of "Internet + government affair" is exogenous (Fig 2, Table 4).

**Table 4:** Parallel trend test.

	estimation Results
trend*treated	0.0123
	(0.54)
edu	-0.146***
	(0.000)
urbanize	-0.538***
	(0.000)
stru_dev	-0.066***
	(0.002)
trend	-0.109***
	(0.000)
open	0.066***
	(0.003)
Spatial fixation effect	Control

Note: p-value is in parentheses.

*, ** and *** indicate significance levels of 10%, 5% and 1%, respectively, the same below.

Source: Calculated by the author.

#### 2. Results of model regression

According to the regression results of SDID, the following conclusions can be drawn:

① DID coefficient is significantly negative, indicating that the direct spillover effect brought by Internet plus policy will narrow the digital divide between regions"Internet + government affairs" policy can accelerate the popularization of infrastructure and improve the overall level of digital government affairs.

② The coefficient of *W*_*NTT*_*DID* is significantly positive, indicating that the experimental group had a positive spillover effect on the adjacent control area. "Internet + government affairs" policy supports experimental group to vigorously build Internet government affairs platform, this kind of infrastructure is mostly used for the communication between vertical government systems, but less for communication and interaction with neighboring cities. The construction of government affairs platform has strong local characteristics, the spillover effect of knowledge and technology is not significant, the digital government affairs level of the experimental group and the adjacent control group cannot be improved in coordination, but the digital gap between them is widened.

③ The coefficient of *W*_*TT*_*DID* is also significantly positive, which indicates that the experimental group also has a positive spatial spillover effect on the adjacent experimental areas. The first situation is that there is a big difference in the maturity of digital government construction on the Internet platform between the two adjacent experimental groups, while the promotion effect of digital government level presented by the same policy is quite different. High-level areas can rely on the existing platforms to rapidly promote the construction of “Internet + government affairs”, while low-level areas need a certain period of time to promote and popularize facilities, finally widening the digital divide. The second situation is that the infrastructure and other aspects of the two neighboring experimental groups have little difference, but the local effect of "Internet + government affairs" policy is prominent, and it is difficult to coordinate the digital government construction synchronously. Therefore, the change of either party will widen the digital divide between the two regions.

④It can be found by comparing the three different effects that the direct effect of “Internet + government affairs” policy is the strongest, the second is the spillover effect between the experimental groups, and the last is the spillover effect of the experimental group to the control group. It shows that the influence of the policy of “Internet + government affairs” on the digital divide is transmitted through the direct effect path (Table 5).

**Table 5:** Spatial DID regression results.

	SDM model	SDM model
	Geographic distance matrix	Economic distance matrix
DID	-5.8192***	-5.8984***
	(0.005)	(0.005)
*W*_*T*,*T*_	4.2863***	3.5825***
	(0.000)	(0.000)
*W*_*NT*,*T*_	3.7857***	3.1089****
	(0.000)	(0.000)
rho	0.72703***	0.7255***
	(0.000)	(0.000)
open	-42.6011***	-42.865***
	(0.000)	(0.001)
edu	11.1379***	
	(0.000)	
lngdppc		6.2686**
		(0.013)

Note: p-value is in parentheses.

*, ** and *** indicate significance levels of 10%, 5% and 1%, respectively, the same below.(III) Benchmark Regression Results and Analysis.

Source: Calculated by the author.

Models (1)–(2) report mixed OLS and fixed-effect model regression results in turn. From the regression results, it can be seen that the digital divide is positively correlated with the income divide. In the mixed OLS model, every 1 unit increase in the digital divide will widen the income divide by 0.1251 units. In the fixed-effect model, every 1 unit increase in the digital divide will widen the income divide by 0.103 units.

Considering that the digital divide index is not an exogenous variable, and there is a possibility of mutual causation between digital divide index and income divide, it is necessary to reduce the error of model parameter estimation through instrumental variable processing. Through the parallel trend test in the first stage regression of spatial DID + IV, the externality of “Internet + government affairs” policy is illustrated. The estimated value of digital divide index based on the counterfactual framework constructed by spatial DID is also exogenous. What’s more, this estimate value cannot directly affect the income disparity between regions, and satisfies the two conditions of correlation and externality of instrumental variables. Hence, the digital divide index estimated by spatial DID regression is selected as instrumental variable in this paper.

Model (3) substitutes the estimated value of the digital divide index obtained from the spatial DID model into the regression, and presents the two-stage regression result of spatial DID + IV. Every 1 unit increase of digital divide will widen the income divide by 0.134 units. After adding the control variables, the F value of the first stage is 20.38, greater than 10, which is in accordance with the rule of thumb, indicating that there is no problem of weak instrumental variables. Then the over-identification test of the externality of the instrument variable was carried out, and the p value was 0.697. It is considered that the selected instrumental variable is exogenous and not related to the disturbance term, which further verifies the rationality of the instrumental variable. After dealing with endogenous issues, the conclusion remains that the digital divide widens income disparities.

Among the control variables, there is a negative correlation between the average length of education and the income divide. The improvement of education level helps to upgrade the gradient of human capital, improves the efficiency of labor resource allocation and promotes the homogeneous convergence of income divide. The improvement of urbanization level narrows the income divide, and the steady progress of urbanization facilitates the free flow of factors, promotes the rural labor force to transfer from the low-return sector to the high-return sector and obtain structural dividends, thus narrowing the income divide between urban and rural areas. There is a positive correlation between regional opening level and income divide. China’s position in the division of labor system of global value chain has moved up gradually from a large trading country to a trading power. The improvement of regional openness puts forward higher requirements for human capital, reduces the marginal efficiency of labor force to a certain extent, and widens the wage divide of employees with different skill levels (Table 6).

**Table 6:** Benchmark regression results.

	pooled OLS	fix effect	IV
*gap*	0.125***	0.103**	
	(0.000)	(0.000)	
$g\hat{a}p$			0.134**
			(0.014)
*urbanize*	-0.534***	-0.096***	-0.522***
	(0.000)	(0.000)	(0.000)
ln *gdppc*	-0.098***	-0.027***	
	(0.001)	(0.000)	
*open*	0.0714***	0.142***	0.1149**
	(0.001)	(0.001)	(0.022)
*stru*_*dev*	-0.073***	0.0001***	
	(0.000)	(0.000)	
*edu*	-0.149***	-0.0399***	-0.0287***
	(0.000)	(0.000)	(0.000)
Time effect	Control	Control	Control
Spatial effect	Control	Control	Control

Note: p-value is in parentheses.

*, ** and *** indicate significance levels of 10%, 5% and 1%, respectively, the same below.

Source: Calculated by the author.

## V. Further analysis

### (I) Robustness test

Benchmark regression leads to the conclusion that the digital divide will widen income divide. In order to test the robustness of the above results and further demonstrate the impact of the digital divide on the income divide, the following three robustness tests will be carried out: First, change the measurement method of dependent variables, and replace the explanatory variables in simple OLS regression with urban-rural income ratio to verify again. The results of model (1) show that the digital divide still has a significant effect on the income divide after changing the indicator of income divide to urban-rural income ratio. Secondly, 5% of the samples with the highest and lowest digital divide index, i.e., Beijing, Guangzhou, Shanghai, Dongguan City, Hangzhou City, Foshan City, Suzhou City, Zhuhai City, Tongren City, Haozhou City, Qinzhou City, Dazhou City, Yulin City, Yongzhou City, Haidong City and Luohe City were removed, and then the robustness test was carried out. Model (2) presents the regression results of this method and it is found that the conclusion that the digital divide widens the income divide is still valid. Concurrently, different measurement methods are used to calculate the digital divide index, and the entropy weight method is replaced by the main component analysis method. Model (3) reports a regression report using principal component analysis to measure the digital divide index, which increases the income divide by 0.323 units for every 1 unit increase of the digital divide.

Through the above three robustness tests, it is seen that the conclusion obtained in the benchmark regression model is valid, showing that the conclusion that the digital divide widens the income divide is robust (Table 7).

**Table 7:** Robustness test.

	Urban-rural income ratio	Elimination of extreme values	Principal component analysis
divide	0.054***	0.081***	0.323***
	(0.001)	(0.002)	(0.000)
open	0.028	0.015	0.001
	(0.169)	(0.567)	(0.000)
edu	-0.105***	-0.157***	-0.174***
	(0.000)	(0.000)	(0.000)
urbanize	-0.092***	-0.564***	-0.599***
	(0.000)	(0.000)	(0.000)
stru_dev	-0.061***	-0.084***	-0.065***
	(0.000)	(0.000)	(0.000)
lngdppc	-0.078***	-0.117***	-0.271***
	(0.000)	(0.000)	(0.000)
_cons	6.025***	1.139***	1.244***
	(0.000)	(0.000)	(0.000)
Time effect	Control	Control	Control
Spatial effect	Control	Control	Control
N	1400	1384	1400
F value	51.21	66.84	69.90
	(0.000)	(0.000)	(0.000)
Adjusted R^2	0.39194	0.4638	0.4405

Note: p-value is in parentheses.

*, ** and *** indicate significance levels of 10%, 5% and 1%, respectively, the same below.

Source: Calculated by the author.

#### (II) Heterogeneity analysis

China’s digital divide presents obvious spatial differentiation, we first divide the samples into groups based on regional distribution (Table 8).

**Table 8:** Results based on regional heterogeneity.

	Eastern Region	Central Region	Western Region
$g\hat{a}p$	0.216***	-0.0155	0.106*
	(0.001)	(0.712)	(0.097)
Control variable	YES	YES	YES
*N*	495	485	420

Note: p-value is in parentheses.

*, ** and *** indicate significance levels of 10%, 5% and 1%, respectively, the same below.

Source: Calculated by the author.

From the results of heterogeneity analysis, it is demonstrated that the influence of digital divide on income divide is significant in the central and eastern regions, but not in the western regions, it is mainly because the form of digital divide changes with the development of digital economy, from traditional "access divide" gradually evolved into the "utilization divide" and "benefit divide". Due to the low digital level in the western region as a whole, the construction of digital infrastructure is slow. The income divide in different regions mainly comes from traditional factors of production, such as capital and labor, and the application of information technology belongs to a kind of skill-biased progress, so the influence of digital divide on income divide is not obvious in western region. The influence of digital divide on income divide is the most significant in eastern China because of the early start of digital economy and obvious difference in digital technology returns (Table 9).

**Table 9:** Results based on coastal areas or inland areas.

	Coastal areas	Inland Areas
$g\hat{a}p$	0.179**	-0.003
	(0.03)	(0.929)
Control variable	YES	YES
*N*	225	1145

Note: p-value is in parentheses.

*, ** and *** indicate significance levels of 10%, 5% and 1%, respectively, the same below.

Source: Calculated by the author.

According to the maritime standard HYT094-2018 code of coastal administrative area, the whole country is divided into coastal areas and inland regions, and the sub-sample regression is carried out. The regression results express that the digital divide in coastal regions has a significant impact on the income divide. An increase in the digital divide by 1 unit increases the income divide by 0.284 units. By virtue of the advantage of natural geographical location, coastal areas attract sufficient funds and advanced technology, and the digital economy develops rapidly. While developing, the difference of digital dividend returns is obvious, so the digital divide has a high impact on income distribution. However, due to the restriction of transportation and other conditions, the development of digital economy is at a relatively low level, and the influence of digital divide on income divide is not obvious (Table 10).

**Table 10:** Different types based on the digital divide.

	Traditional Digital Divide	New types of Digital Divide
$g\hat{a}p$	0.136***	0.155***
	(0.000)	(0.000)
Control variable	YES	YES
*N*	1400	1400

Note: p-value is in parentheses.

*, ** and *** indicate significance levels of 10%, 5% and 1%, respectively, the same below.

Source: Calculated by the author.

We divide the digital divide into "access divide", "use divide" and "benefit divide" in the process of index construction. The former two focus on the construction of digital infrastructure and are therefore unified into the traditional digital divide. The "benefit divide" is defined as a new digital divide. According to the regression results, the impact of the new digital divide on income distribution is higher than that of the traditional digital divide. Every 1 unit increase in the new digital divide will widen the income divide by 0.170 units. The spread of mobile technology gradually bridges the traditional digital divide, while the new digital divide has been widened due to the difference between the growth and iteration effects of the digital economy. The digital divide is manifested more prominently by the difference in economic benefits brought by the Internet, so the new digital divide has a more significant impact on the income divide.

### (III) Mechanism verification

The digital divide will widen the income divide of Chinese residents. For the purpose of better explaining the relationship between the two, the transmission mechanism behind it will be further discussed from three perspectives of information asymmetry human capital differentiation and industrial structure upgrading.

In order to test the information asymmetry effect caused by the access divide, we introduce the interaction between post and telecommunication service income (post) and digital divide index. The first column of the regression results reports the results of the model, and the interaction between the digital divide index and post and telecommunication revenue is significantly positive, expressing that narrowing the digital divide can perfect income distribution by reducing information asymmetry. In regions with large access divide, the phenomenon of information asymmetry is obvious. In the regions with low digital development level, the low availability and high search cost of information resources restrict the improvement of local income level.

In order to test the effect of the human capital differentiation caused by using the divide, we introduce the interaction term between laborpolar variable and digital divide index. The third column of regression results reports the results of the model, where the interaction between the digital divide index and the share of the tertiary labor force is significantly positive. The labor market polarization and human capital differentiation brought by informatization and digitization will further widen the income divide between individuals.

In order to test the delayed effect of industrial upgrading caused by the benefit divide, we introduce the interaction term of proportion of labor in tertiary industry (tratio) and digital divide index in the tertiary industry. The third column of the regression results reports the results of the model, where the interaction between the digital divide index and the share of the tertiary sector labor force is significantly negative, showing that by narrowing the digital divide, the optimization and upgrading of industrial structure can be promoted, thus reducing the income divide. The expansion of the digital divide will slow down the speed of industrial diffusion, which is mainly due to the high threshold of digital industry, which makes it difficult to spread to areas with low technological level. Also, it is not conducive to the free movement of labor force between different regions and industries. Some studies show that the application of information technology increases the income share of capital and technology and reduces the income share of labor, while the income of the people in the backward areas is mainly derived from labor, which exacerbates the income inequality between regions. Narrowing the digital divide can improve income distribution through two channels. The first is factor allocation effect, which can promote labor transfer to regions or industries with high rate of return, improve the skill level of laborers and reduce income divide. The second is knowledge spillover effect. The existence of digital divide affects the dissemination of knowledge technology and creates an invisible barrier. After breaking the barrier, the diffusion of digital knowledge and technology can be accelerated so that the development of regional integration can be realized (Table 11).

**Table 11:** Mechanisms test.

	Asymmetric Information Mechanism	Differentiation Mechanism of Human Capital	Upgrading Mechanism of Industrial Structure
$g\hat{a}p$	0.139**	0.199***	0.191***
	(0.014)	(0.000)	(0.000)
lngap*post	0.0969***		
	(0.001)		
gap*laborpolar		0.0202***	
		(0.000)	
gap*tratio			0.0179***
			(0.000)
*post*	-0.0742	0.119**	0.119**
	(0.440)	(0.030)	(0.028)
*laborpolar*	0.124	-2.832**	-0.449**
	(0.946)	(0.022)	(0.093)
*tratio*	0.129***	0.116***	0.0876***
	0.000	0.000	(0.002)
*open*	0.106**		
	(0.041)		
*stru_dev*	-0.0000619**	-0.0000593**	-0.0000588**
	(0.036)	(0.044)	(0.046)
*edu*	-0.0319***	-0.0294***	-0.0317***
	(0.000)	(0.000)	(0.000)
*urbanize*	-0.488***	-0.487***	-0.489***
	(0.000)	(0.000)	(0.000)
_cons	-0.141	-0.297	-0.236
	(0.642)	(0.229)	(0.338)
Time effect	Control	Control	Control
Spatial effect	Control	Control	Control
R^2^	0.393	0.393	0.393
*N*	1400	1400	1400

Note: p-value is in parentheses.

*, ** and *** indicate significance levels of 10%, 5% and 1%, respectively, the same below.

Source: Calculated by the author.

## VI. Conclusions

This paper uses the panel data of 280 prefecture-level cities in China from 2014 to 2018 to explore the relationship between digital divide and income gap. Different from the previous studies, we focus on the benefit divide. In addition, it focuses on the mechanism and heterogeneity of income distribution influenced by the digital divide. It is found that: (1) The existence of digital divide widens the income divide between regions, and each increase in the digital divide by 1 unit widens 0.134 units. (2) The influence of "Internet + government affairs" policy on the digital divide has both direct and indirect effects, and the local effect of policy implementation on the digital divide is the most obvious. (3) Information asymmetry, differentiation of human capital and upgrading of industrial structure have intermediary effects, which play an important role among access, use and benefit divide respectively. (4) After considering the regional differences, it is learned that the digital divide in the eastern coastal areas has more significant impact on the income divide. After considering the differences of digital divide at different levels, it is found that the new digital divide has the most obvious effect on widening the income divide.

Considering that the digital divide can affect income distribution through three channels: access, use and benefit divide, we put forward some suggestions for each mechanism. First of all, access divide will bring information asymmetry effect, so it is necessary to improve infrastructure construction to reduce information asymmetry effect. Digital divide in essence, is a kind of information divide. The key to bridging the digital divide is to broaden the availability of information, widen the channel of information transmission and optimize the information communication environment. Secondly, it is also necessary to strengthen the construction of digital access capacity in backward areas through new infrastructure construction, promote the development of information infrastructure towards inclusion, and empower the indexation growth of economy. Thirdly, access divide will bring about the effect of differentiation of human capital, so it is necessary to elevate the digital degree of human capital, perfect the non-digital policy system, and consummate the human capital from "quantity" to "quality" by the influence of digital technology spillover in the backward area, and lowerdown the income inequality between digital-intensive area and non-digital-intensive area through technology complementation. Moreover, the improvement of non-digital policy embodiment can reduce the damage brought by high monopoly to the fair competition market environment. Finally, the benefit divide will bring the sluggish effect of industrial upgrading, so the suggestion of adjusting industrial structure and releasing "digital" dividend is put forward. We will establish a mechanism for inter-regional talent flow, enhance the spillover effect of knowledge and technology, and coordinate inter-regional innovation capacity. Meanwhile, we will actively promote the deep integration of digital economy and real economy based on Internet of Things, 5G, artificial intelligence and other new technologies, accelerate the combination of data elements and traditional production factors, exert the resultant force of industrial digitalization and digital industrialization to boost the rationalization and upgrading of industrial structure and build a new development pattern.

## Supporting information

S1 Data (DO)

S2 Data (DTA)

S3 Data (DTA)

S4 Data (DTA)
